# Tactile Sensitivity of Women with Turner Syndrome

**DOI:** 10.3390/ijerph16203870

**Published:** 2019-10-12

**Authors:** Julia Jajor, Anna Kostiukow, Włodzimierz Samborski, Elżbieta Rostkowska, Aleksandra Śliwa, Katarzyna Antosiak-Cyrak

**Affiliations:** 1 Department of Rheumatology and Rehabilitation, Poznan University of Medical Sciences, 28 Czerwca 1956r. Street 135/147, 61-545 Poznan, Poland; julia.jajor@gmail.com; 2 Department of Rheumatology and Rehabilitation, Poznan University of Medical Sciences, 28 Czerwca 1956r. Street 135/147, 61-545 Poznan, Poland; annakostiukow@ump.edu.pl (A.K.); samborskiw@o2.pl (W.S.); 3 Department of Biomedical Basis of Physiotherapy, University of Computer Sciences and Skills, 17A Rzgowska Street, 93-008 Lodz, Poland; jaroe8@wp.pl; 4 Department of Cell Biology, Poznan University of Medical Sciences, 5D Rokietnicka Street, 60-806 Poznan, Poland; glodek@ump.edu.pl; 5 Laboratory of Swimming and Water Lifesaving, Poznan University of Physical Education, Krolowej Jadwigi 27/39, 61-871 Poznan, Poland

**Keywords:** turner syndrome, tactile sensitivity, semmes-weinstein aesthesiometer

## Abstract

Physical manifestations of Turner syndrome include short stature, a webbed neck, and a shield chest with widely spaced nipples. An aspect of the disease which has not been sufficiently explored so far is the tactile sensitivity of Turner syndrome patients. Thus, the aim of the study was to assess the threshold of tactile sensitivity on hands and feet of women suffering from Turner syndrome. Information on the participants of the study was collected on the basis of questionnaires, as well as anthropometric measurements using a skinfold caliper. Semmes-Weinstein Aesthesiometer was used to find the tactile sensitivity threshold of hands and feet of study participants. Based on the results of the study, significant differences in tactile sensitivity between women with Turner syndrome and healthy women were found. Affected women seem be more sensitive to the touch on the feet than healthy volunteers. The results of the study showed that the tactile sensitivity of women with Turner syndrome is different from that of healthy women.

## 1. Introduction

Turner syndrome affects one in 2500 live female births and is caused by a complete or partial loss of one X chromosome. Half of the individuals suffering from the condition have a monosomy X karyotype, while the other half is characterized by mosaicism.

Newborns affected by Turner syndrome have lower body weight and are shorter compared to healthy infants. Other physical manifestations which are seen in girls with Turner syndrome include skeletal disproportions, such as micrognathia, high-arched palate, short fourth metacarpals, genu valgum, Madelung wrist deformities, and short limbs.

Since growth is usually compromised in untreated patients with Turner syndrome, affected girls have an average final height of 143 cm, which is about 20 cm lower compared to the healthy population [1]. Early treatment with growth hormone and oxandrolone usually leads to a substantial increase in adult height. Studies involving Turner syndrome patients indicate that a higher final growth is achieved in girls who have a more favorable ratio of the torso length to the length of the lower limbs. Studied patients who exhibited greater deficit of leg length and increased trunk to leg length ratio were characterized by lower final height [2]. Still, even after treatment with recombinant growth hormone, girls with Turner syndrome achieve lower height than their healthy peers [3]. It is postulated that the reason for this discrepancy in these cases is reduced sensitivity to growth hormone of Turner patients rather than its deficiency [4,5]. Nonetheless, the hormone has beneficial effects on body composition of the patients. Growth hormone is said to increase lean body mass and reduce the tendency to accumulate adipose tissue [6].

Turner syndrome patients are at risk of congenital malformations of the heart and renal abnormalities. Ovarian dysgenesis and premature ovarian failure in Turner syndrome are associated with the lack of properly developed secondary sex characteristics, infertility, and osteoporosis. However, sex hormones deficiency may also lead to increased cardiovascular risk, diabetes, autoimmune disorders, and influences the state of physical fitness. Since research evidence supports the effectiveness of pubertal estrogen replacement with E2 in Turner syndrome most patients undergo this regimen [7].

Studies show that Turner syndrome girls perform worse on spatial, attentional, and memory cognitive tasks and have more psychosocial problems than short-stature girls with a normal karyotype. It is postulated that estrogen may act as a neuro-protector, which may selectively benefit certain cognitive tasks. Thus, deficiencies in estrogen, like the ones experienced by Turner syndrome patients can result in impairment of cognitive abilities [8].

Among the many impaired factors, senses are included: Touch, sight, hearing, smell, and taste determine human cognitive abilities, starting in infancy. Thus, the impairment of any of the senses, including the sense of touch, reduces cognitive abilities. What is more tactile sensitivity is a feature that can significantly support the performance of daily motor coordination tasks and human’s motor development which improves body composition and physique. Tactile sensitivity of hands and feet can also profoundly influence physical fitness and affect the overall quality of life.

We believe that tactile sensitivity of Turner syndrome patients is an aspect of the disease, which has not been sufficiently explored. Especially in the light of studies showing how important sensory integration is in treatment of children with developmental and behavioral disorders. This article is the first that documented the phenomenon of changes in tactile sensitivity in women suffering from Turner syndrome.

Thus, the aim of the study was to assess the threshold of tactile sensitivity on hands and feet of patients suffering from Turner syndrome compared to heathy female volunteers.

The study also verified whether growth hormone therapy, the use of hormone replacement therapy, body fat, height, and body weight of patients have an impact on the threshold of tactile sensitivity.

## 2. Materials and Methods

The study group consisted of 30 women with Turner syndrome (group T), while the control group (group Z) included 37 healthy female volunteers not affected by the condition. Women from the control group were of similar age and lived in similar social conditions to those with Turner syndrome.

Information about the research was disseminated among Turner syndrome support societies and participants were recruited during support meetings. The inclusion criteria for the study were the following: Females diagnosed with Turner syndrome, 10–70 years old, with no history of diabetes. After completing the screening process which determined patients who met the inclusion criteria, the potential participants had a consultation and were provided with all information about the study. Written informed consent for the study was obtained from all participants or their legal guardians. The control group was recruited among healthy female individuals, 10–70 years old, who were not diagnosed with Turner Syndrome and did not suffer from any diseases associated with growth.

The exclusion criteria were blindness and visual impairment of participants as well as severe intellectual disability and mental disorders preventing proper verbal contact.

The study was approved by the Bioethical Committee of the Poznan University of Medical Sciences (Resolution No. 517/14).

Measurement of the tactile sensitivity threshold of hand and feet was carried out with the use of Touch-Test™ Sensory Evaluators (Stoelting Co., Wood Dale, USA) according to the Semmes-Weinstein Monofilaments Test.

Tactile sensitivity was measured using Semmes-Weinstein Aesthesiometer on both hands and both feet. The measurement points were the little finger, the index finger, and the metacarpus of the hands; on the toe, the external edge of the foot at the small toe, and the outer edge of the foot in the middle of its length in case of feet.

During the test the subject was lying on their back with their eyes closed. Aesthesiometer’s filaments were applied perpendicularly to the above-mentioned measurement points. The thinnest filament which exerted a tactile impression determined the threshold of the subject’s tactile sensitivity [9]. The numerical result of the test was the value, indicated on filament in a range 1.65 to 6.65. Each value of sensitivity threshold corresponds to a specific target force. Examined places on the skin were tested by applying successive filaments from the thinnest to the thickest. The lower the numerical value and thus the target force, the better the result of the test. Tactile sensitivity for healthy individuals according to the Touch-Test™ Sensory Evaluators ranges from 1.65 to 2.83 [10].

Tactile sensitivity as a human sense depends on the distribution of touch receptors on the skin and the depth of their location on the skin. If the skin is thick, the receptors are deeper and thus the researcher will find a higher tactile sensitivity threshold what means a weaker response to touch. Nevertheless, the researcher noted the specific reaction of the examined person either “I feel the touch of the measuring device” or “I do not feel the touch”. Therefore, testing the threshold of tactile sensitivity using the Semmes-Weinstein aesthesiometer gives precise results appropriate for statistical analysis. However, the impact of various factors on tactile sensitivity was not studied, but differences in tactile sensitivity between women with Turner syndrome and healthy women were researched. Researcher examined correlations of tactile sensitivity with the thickness of skinfolds, the use of growth hormone, the use of replacement therapy, and the level of education.

A skinfold caliper was used to measure the fatness of the body. Participants were examined in a standing position, with upper limbs hanging freely along the torso and eyes directed forward. The researcher, using his fingers, grabbed a given skinfold and measured its thickness with a caliper. The thickness of the following skinfolds was assessed: Folds on the cheek, folds on the chin, axillary folds, folds on the back and the front of the arm, shoulder folds, folds on the tenth rib, folds on the abdomen, folds on the side of the trunk, kneecap folds, and popliteal folds. Anthropometric measurements included also the assessment of height and weight of the participants.

All women were subjected to a questionnaire in which personal data and information regarding growth hormone treatment and hormone replacement therapy and accompanying diseases were collected.

All obtained data was analyzed using Statistica 13.3 software (TIBCO Software Inc., Palo Alto, USA). Due to the lack of normal distribution of the data, non-parametric statistics were applied. Differences between the two studied groups were analyzed using Mann-Whitney U test, while associations between variables in the studied groups of Turner syndrome patients and healthy volunteers were assessed using Spearman’s rank correlations. *p* < 0.05 was considered to indicate statistical significance.

## 3. Results

### 3.1. Turner Syndrome Patients Differ from Healthy Women of the Same Age in Terms of Height and Weight

The age of patients with Turner syndrome (group T) was between 10 and 61 years, while women in the control group were aged between 17 and 68. No difference in the age of the analyzed groups of women was detected (Table 1).

Of all women with Turner syndrome taking part in the study, 12 had never been treated with growth hormone; the remaining 17 had been or were being treated with the hormone. One patient did not answer the question about the use of growth hormone.

The examined groups of women showed differences in height and body weight (Table 2).

### 3.2. Women with Turner Syndrome Have the Same Tactile Sensitivity of Hands as Healthy Women

**Figure 1:**
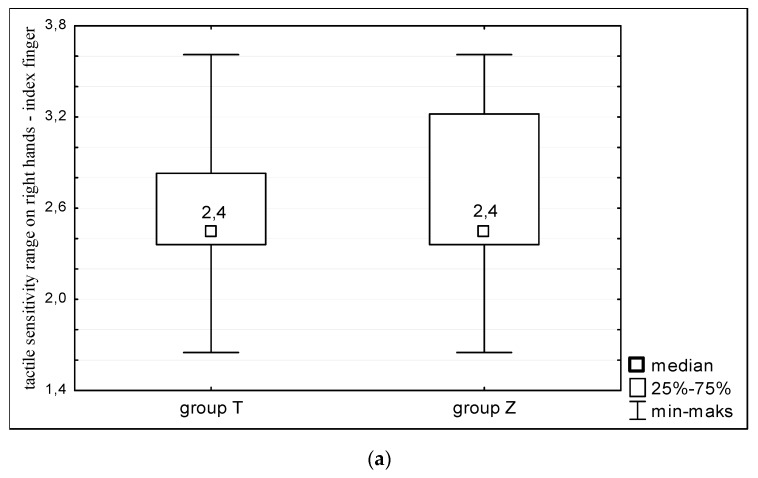

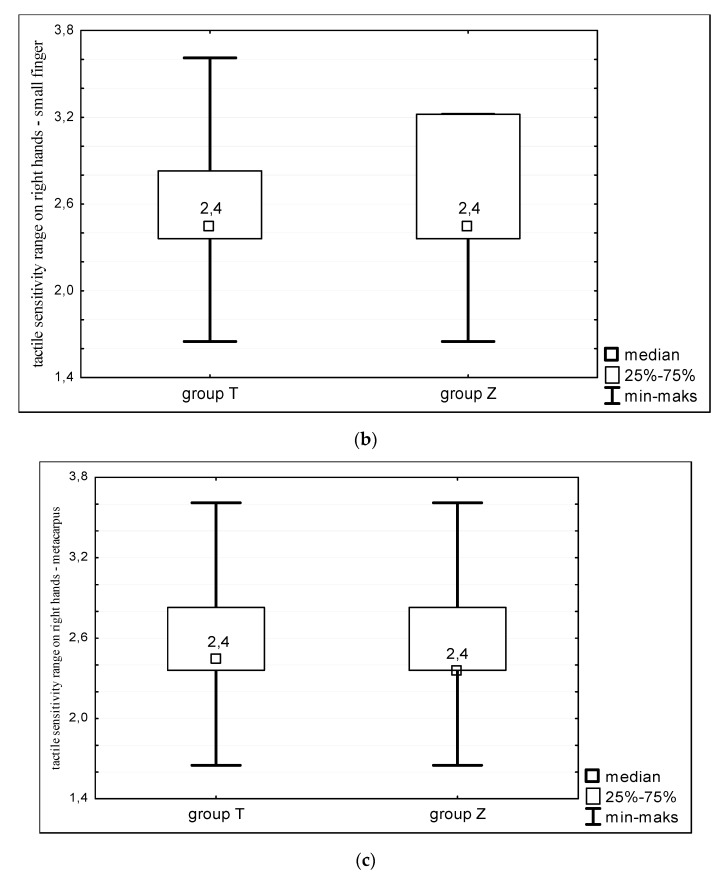
Tactile sensitivity of right hands of women with Turner syndrome (group T) and healthy women (group Z). Sensitivity measurements taken at: (**a**) the index finger; (**b**) small finger; and (**c**) metacarpus.

**Figure 2:**
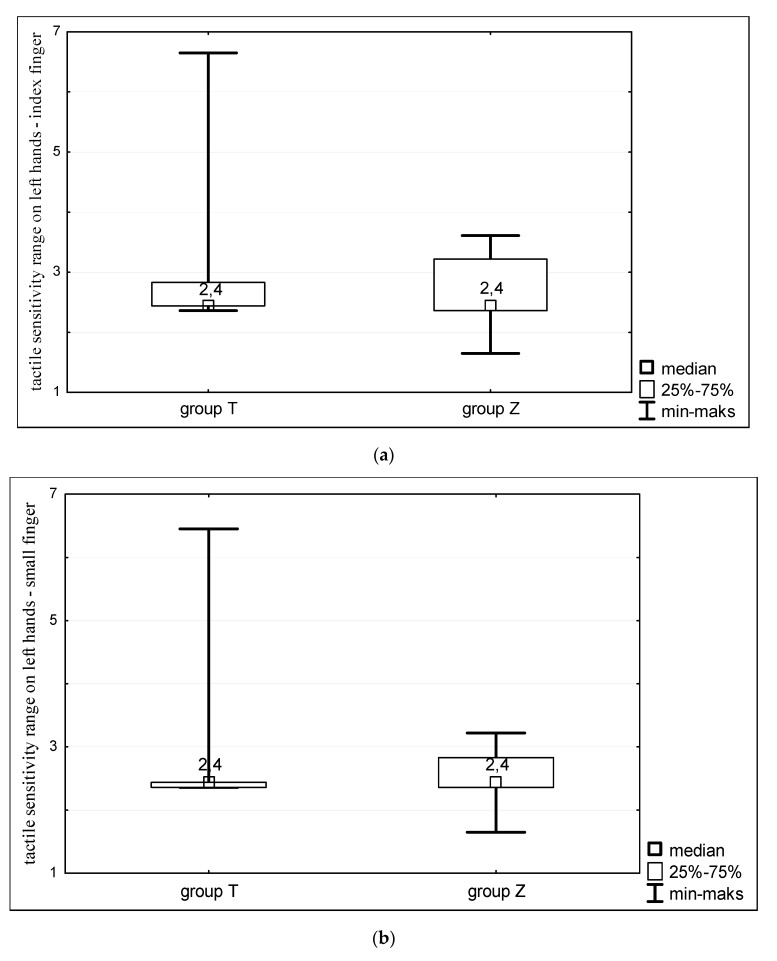

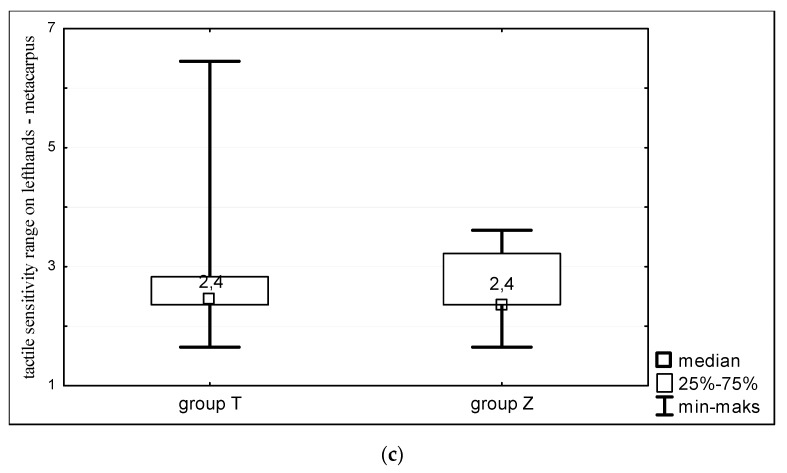
Tactile sensitivity of left hands of women with Turner syndrome (group T) and healthy women (group Z). Sensitivity measurements taken at: (**a**) the index finger; (**b**) small finger; and (**c**) metacarpus.

The study showed that there are no significant differences in terms of tactile sensitivity of hands between women with Turner syndrome and heathy women. All studied women showed similar sensitivity to the touch on both right (Figure 1) and left hands (Figure 2). The mean value of sensitivity threshold for all analyzed points on hands in both groups corresponded to 0.04 g of target force, which is within the range of sensitivity typical to healthy individuals (Figure 1 and Figure 2).

### 3.3. Women with Turner Syndrome Have Different Tactile Sensitivity on the Feet than Healthy Women

The analysis of the results obtained in the Semmes-Weinstein Monofilaments Test conducted on the study participants showed differences in tactile sensitivity of selected points on the feet between the group of Turner syndrome women and healthy volunteers.

**Figure 3:**
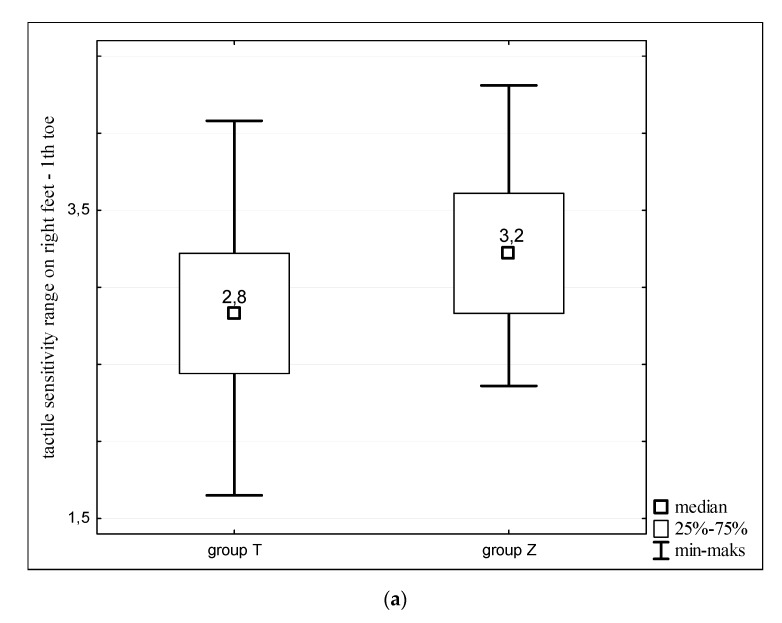

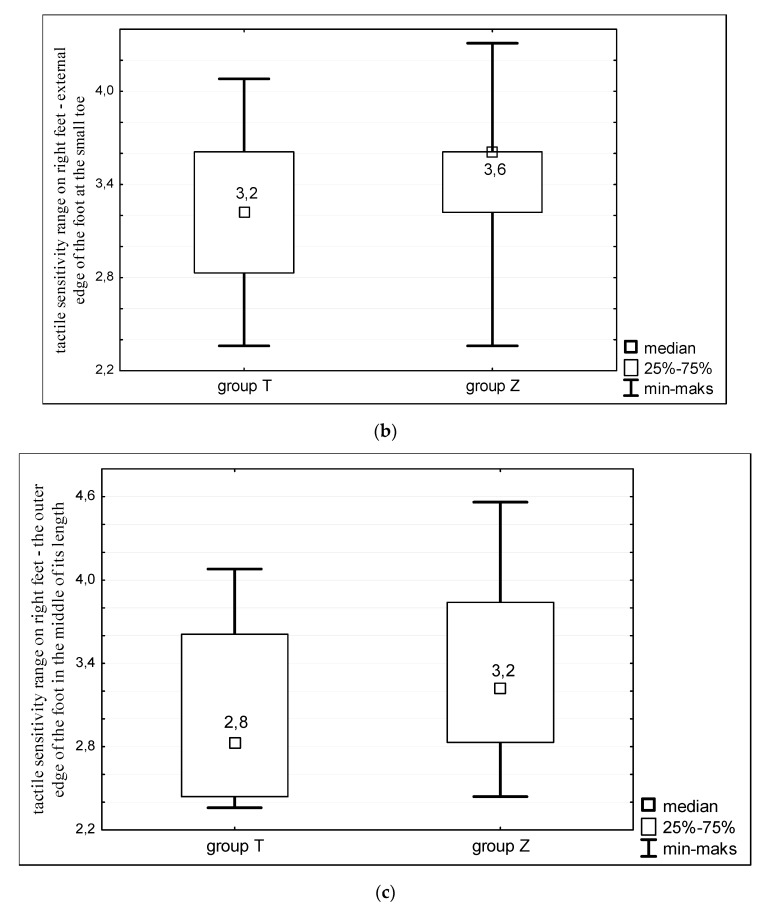
Tactile sensitivity of right feet of women with Turner syndrome (group T) and healthy women (group Z). Sensitivity measurements taken at: (**a**) the first toe; (**b**) external edge of the foot at the small toe; and (**c**) the outer edge of the foot in the middle of its length.

Individuals with Turner syndrome showed higher tactile sensitivity of the right foot in the measurement point of the first toe and the external edge of the foot at the small toe than healthy women (Figure 3). In both cases differences were statistically significant (*p* < 0.05). The tactile sensitivity threshold of the point identified at the outer edge of the right foot in the middle of its length was the same for both studied groups.

**Figure 4:**
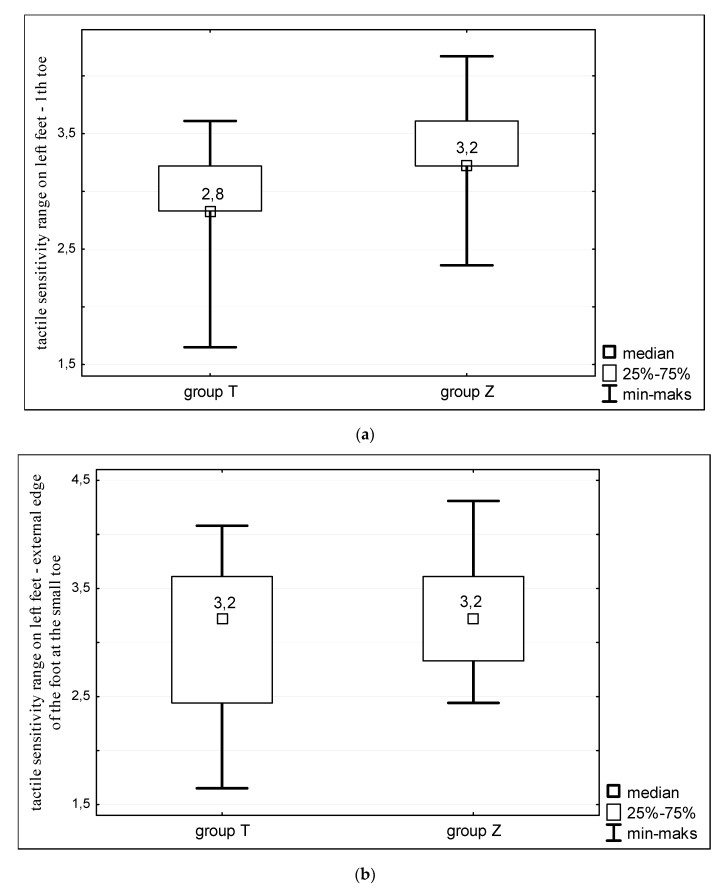

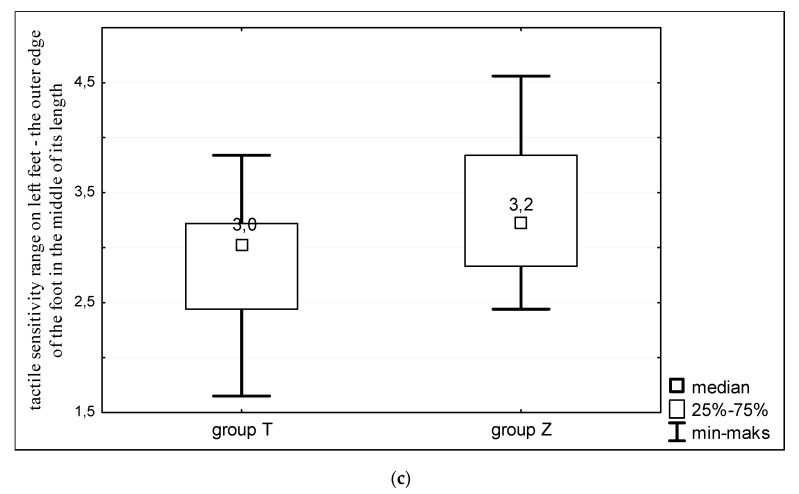
Tactile sensitivity of left feet of women with Turner syndrome (group T) and healthy women (group Z). Sensitivity measurements taken at: (**a**) the first toe; (**b**) external edge of the foot at the small toe; and (**c**) the outer edge of the foot in the middle of its length.

The left feet of the studied women also showed differences in tactile sensitivity. Statistically significant differences were noted in the sensitivity threshold of the first toe (*p* < 0.001) and in the middle on the outer edge of the left foot (*p* < 0.05) (Figure 4). In all cases sensitivity of the feet was greater in Turner syndrome patients.

The data obtained in tactile sensitivity tests showed associations with results obtained in body structure measurement of the participants. In both groups, tactile sensitivity showed statistically significant negative correlation with the thickness of selected skinfolds (Table 3).

### 3.4. Changes in Tactile Sensitivity of Women with Turner Syndrome May be Associated with Hormone Treatment

The results obtained from the comparison of tactile sensitivity threshold between women with Turner syndrome who underwent growth hormone stimulation with those who did not undergo such treatment showed statistically significant differences in selected points of the hands and feet.

Women within the T group who were treated with the growth hormone were more sensitive to the touch on the index finger (*p* < 0.05), the small finger (*p* < 0.05), and on the metacarpus of the right hand (*p* < 0.05), as well as on the outer edge of the right foot at the small toe (*p* < 0.05) (Table 4).

The analysis of estrogen replacement therapy effect on tactile sensitivity showed that there are differences in sensitivity in the case of the left foot. Women with Turner syndrome undergoing hormone replacement therapy were less sensitive to the touch on their left feet than women from group T who did not take estrogen. Differences were noted for all three measurement locations: On the first toe, outer edge at the small toe, and on the outer edge in the middle of its length and were all statistically significant (*p* < 0.05) (Table 5).

In the group of Turner syndrome women, those who had higher education turned out to be less sensitive on left feet on the first toe, in comparison with women with low education (Table 6).

## 4. Discussion

Touch is the first of senses to develop in a human being and it provides the body with a sensory scaffolding. However, tactile perception is in truth a part of a complex human sensory system, consisting of proprioception, mechanoreception, thermoreception, and nociception. Studies show also that tactile sensitivity supports the performance during movement tasks.

Among many physical manifestations, tactile sensitivity of women with Tuner syndrome has not received much attention and our study is the first to document changes in tactile perception of hands and feet of Turner syndrome patients in comparison to healthy female volunteers.

The Semmes-Weinstein Monofilaments Test did not show any statistically significant differences in tactile sensitivity of the hands between groups. However, such differences were found on feet. What is more, a correlation between thickness of skinfolds and the threshold of tactile sensitivity in patients was found, which proves that higher fatness in the upper body was associated with reduced tactility on the feet.

Ontogenetic development of tactile sensitivity depends on many genetic and environmental factors [11,12]. It has an impact on cognitive processes, and therefore affects the quality of life, physical fitness and the general functioning of people. Kozłowska confirmed a positive correlation between the level of education and the level of tactile sensitivity in the group of 1500 individuals aged 7–85 years of both sexes [13]. A similar study was conducted by Kaluga et al., in which group examined 99 women, 19 to 87 years old, with rheumatic diseases and did not find such a correlation. Only the control group, consisting of healthy women aged 23–80, showed such dependence [14]. In our study, a similar analysis was undertaken for patients with Turner syndrome. In this group of patients, however, a different result was obtained than in the group examined by Kozłowska, because those with higher education had poorer tactile sensitivity on the left first toe (the correlation was low).

In other studies of Kaluga et al., it was proven that the type of sports discipline practiced also influences the threshold of tactile sensitivity. These studies have shown that individuals who practice swimming have a decreased threshold of tactile, while basketball players exhibit an opposite phenomenon [15].

Hennig and Sterzing examined the threshold of tactile sensitivity in 30 places on the plantar and dorsal sides of feet using Semmes-Weinstein Monofilaments. The research was conducted in a group of women and men aged 20–35. The plantar side of the feet turned out to be more sensitive [16]. In these studies, there was no comparison of the dorsal and plantar sides of the feet. However, it was shown that in comparison to the palmar side, the plantar side of the feet was more sensitive in women with Turner syndrome. In healthy women this difference was not found.

The level of tactile sensitivity may be influenced by various factors, including biological ones (body weight, age, height, etc.), psychic (intelligence level), or social. According to Kozłowska, the body dimensions may be related to the tactile sensitivity. For example, individuals with low height, have a higher receptor distribution on a given surface unit. Similarly, assuming that higher tactile sensitivity is associated with a higher density of receptors, leaner people will be more sensitive to the touch than obese people [13]. In our studies, this co-occurrence was partially confirmed because women with Turner syndrome turned out to be more sensitive on their feet. The feature that affected the test result was lower body weight, despite greater body fatness. Women with Turner syndrome are characterized by shorter feet, which results in a denser distribution of receptors, which allows them to be more sensitive on the feet. Our research confirmed this difference. Thus, it can be concluded that there are many factors that modify tactile sensitivity.

The conducted studies showed that treatment with growth hormone had a positive effect on the feeling of touch of upper limbs. Patients who used this hormone showed greater tactile sensitivity on right hands. As far as we know there were no other studies documenting the relationship between growth hormone therapy and tactile sensitivity. Other researchers who analyzed tactile sensitivity in people aged 19–88 also did not show statistically significant relationships between the use of medication and the threshold of tactile sensitivity [17].

The results of this research confirmed that the use of hormone replacement therapy affected patients’ tactile sensitivity of their left feet. The effect was negative. A direct relationship between these traits has not been established so far, but the results of studies in 32 women prove that there is a connection between lower tactile sensitivity and estrogen deficiency, which is treated with hormone replacement therapy [18]. What is more, studies confirm that even during the menstrual cycle low estrogen levels correlate with high tactile thresholds [19]. Therefore, we verified whether similar changes occur in Turner syndrome, who suffer from estrogen deficiency.

In further research it should be considered what the differences in feet and hand sensitivity are caused by.

## 5. Conclusions

First of all, the obtained results have a status of basic research. A completely new knowledge about the tactile sensitivity of women with Turner syndrome have been acquired.

Surprisingly we learnt that women with Turner syndrome are more tactile on the feet than healthy women. This is a feature for practical use, they can be more effective in developing body balance. Tactile sensitivity allows to feel the pressure of the feet on the ground and unevenness of the ground, significantly helps the body balance in standing and in motion. This can be a clue for the direction of professional or sports development, and for those who are less physically fit to choose exercises during physical rehabilitation.

We learnt that tactile sensitivity of women with Turner syndrome is similarly conditioned as in the population studies of other authors: Slim and more educated individuals are more tactile sensitive.

Women with Turner syndrome treated with growth hormone are more tactile and this should be an additional argument for using this treatment. What is more Turner syndrome patients treated with estrogen replacement therapy were less touch sensitive. This is certainly not an argument for suspending such treatment but it is a very interesting information. The obtained result can act as a basis for further research where such correlation would be thoroughly explored.

The results of the study confirmed that Turner syndrome patients are characterized by lower stature and greater weight compared to healthy women. It is the first study to document that female individuals with Turner syndrome are more sensitive to the touch on their feet than healthy women. Based on the conducted research, it may be also postulated that among Turner syndrome patients’ differences in tactile sensitivity may be associated with estrogen and growth hormone therapy.

## Figures and Tables

**Table 1:** Age and results of anthropometric measurements in studied groups.

Group T (N = 30)				Group Z (N = 37)			
mean	min–max	SD	v%	mean	min–max	SD	v%
**Age (years)**							
29.1	10–61	14.3	49	33.9	17–68	15.0	44
**Height (cm)**							
147.7	129–169	8.8	6	166.6	152–186	8.1	5
**Weight (kg)**							
54.9	35–79	10.4	19	63.7	46–89	10.1	16

**Table 2:** Comparison of somatic features between groups.

Variable	Mann-Whitney U Test Results	
	Z	P
Weight	−3.2	0.001
Height	−6.3	0.000

**Table 3:** Statistically significant correlations of the tactile sensitivity threshold on hands/feet and the thickness of skinfolds in the group of Turner syndrome women (group T) and healthy volunteers (group Z).

Tactile Sensitivity			Body Building Features	R Spearman	*p*
Body Part	Side	Point	Skinfold		
group T					
hand	left	metacarpus	shoulder	0.39	0.032
			kneecap	0.42	0.020
foot	right	outer edge of the foot in the middle of its length	front of arm	0.37	0.046
foot	left	1th toe	back of arm	0.39	0.035
			front of arm	0.45	0.013
			shoulder	0.41	0.023
group Z					
hand	right	index finger	chin	0.37	0.026
			front of arm	0.39	0.018
hand	left	index finger	chin	0.35	0.036
			front of arm	0.38	0.022
		small finger	chin	0.40	0.015
			front of arm	0.36	0.033
			side of the trunk	0.33	0.047
		metacarpus	front of arm	0.35	0.035
foot	right	1th toe	axillary	−0.56	0.000
		external edge of the foot at the small toe	chin	0.48	0.003
			shoulder	0.33	0.044
		outer edge of the foot in the middle of its length	chin	0.45	0.006
			front of arm	0.36	0.027
foot	left	1th toe	chin	0.35	0.032
		external edge of the foot at the small toe	chin	0.53	0.001
			front of arm	0.47	0.003
		outer edge of the foot in the middle of its length	chin	0.43	0.009
			front of arm	0.35	0.031

**Table 4:** Statistically significant differences in tactile sensitivity among women with Turner syndrome treated and untreated with growth hormone.

Figure	Mann-Whitney U Test Results		
	Women Treated with Growth Hormone	Z	*p*
threshold of tactile sensitivity on index finger, right hands	better	1.97	0.049
threshold of tactile sensitivity on small finger, right hands	better	2.54	0.011
threshold of tactile sensitivity on metacarpus, right hands	better	2.59	0.010
threshold of tactile sensitivity on external edge of the foot at the small toe, right feet	better	2.04	0.042

**Table 5:** Statistically significant differences in tactile sensitivity among women with Turner syndrome treated with estrogen replacement therapy and non-treated group.

Measurement Point	Mann-Whitney U Test Results	
	Z	*p*
left foot—1th toe	3.2	0.001
left foot—outer edge at the small toe	2.1	0.032
left foot—outer edge in the middle of its length	2.2	0.030

**Table 6:** Statistically significant relationships between the threshold sensitivity test and the level of education in group T.

Variables	R Spearman	*p*	Women with Higher Education
group T			
threshold of tactile sensitivity on first toe, left feet	−0.36	0.048	worse
